# 

**Published:** 1867-11

**Authors:** 


### FINANCIAL.

WITH the next issue of the REGISTER, which will be the closing number of Vol. XXI., we shall send out bills to all who are in arrears for subscription; and we hope that all such will be ready and respond promptly.

The sums are small, we know, especially to those who owe them, but in the aggregate very large to us, and the REGISTER very much needs these sums. And indeed, we will suggest that no one who knows himself to be indebted need wait for the bill, but anticipate its coming by sending the amount. There are a few who owe us such sums on subscription that we are unwilling to have them larger until they are paid. A hint to the wise is sufficient.

### THE LAKE ERIE DENTAL ASSOCIATION.

### DENTAL MEETING.

THE fifth annual meeting of the Central States Dental Association will be held in the City of Louisville, Ky., commencing on Thursday, December 26th, 1867, at 10 o'clock, A. M.

This will be the most important meeting of the Association, besides being the most interesting one. I am assured that there will be present several leading Dentists of New York, Philadelphia, St. Louis, and other places. It is to be hoped that all who can possibly do so will be present.

W. H. SHADOAN, *Secretary.*

### OHIO COLLEGE
OF
### DENTAL SURGERY.

The Twenty-second Annual Session of the Ohio College of Dental Surgery will commence on Tuesday the 15th of October, and continue five months.

### FACULTY.

JAMES TAYLOR, M. D., D. D. S.,
Emeritus Professor of Institutes of Dental Science.

C. KEARNS, M. D.,
Professor of Anatomy.

C. W. SPALDING, D. D. S.,
Professor of Histology and Physiology.

GEORGE WATT, M. D., D. D. S.,
Professor of Pathology and Therapeutics,

S. P. CUTLER, M. D., D. D. S.
Professor of Chemistry and Microscopy.

J. TAFT, D. D. S.,
Professor of Operative Dentistry and Dental Hygiene

H. R. SMITH, M. D., D. D. S.,
Professor of Mechanical Dentistry and Metallurgy.

W. T. ARRINGTON, D. D. S.
Professor of Clinical Dentistry.

WILL TAFT, D. D. S.
Demonstrator of Anatomy.

### TEXT BOOKS.

Gray's and Wilson's Anatomy; Dalton's or Carpenter's Physiology; Williams' Pathology; Fown's, Turner's, Graham's, or Brandt and Taylor's Chemistry; Taft's Operative Dentistry; Richardson's Mechanical Dentistry; Harris' Principles and Practice of Dental Science, Tomes' Dental Surgery.

### TERMS OF ADMISSION.

Tickets must be procured at the beginning of the session.

| | |
|---|---|
| Tickets for the course, | $100 00 |
| Demonstrator's Ticket for Anatomy, | 5 00 |
| Matriculation Fee, but once, | 5 00 |
| Diploma Fee, | 30 00 |

### TERMS OF GRADUATION.

Two courses, (including one of dissections,) with a satisfactory dental pupilage.

Eight years reputable practice will be received as equal to one course.

An acceptable thesis upon some subject on Dental science.

Must possess a good moral character, and be twenty-one years old.

For furthei information, address J. TAFT, *Dean*.
238 West Fourth Street.

AMERICAN DENTAL ASSOCIATION—Meets annually (this year at Niagara,) *Pres.* A. Lawrence of Lowell, Mass., *Rec. Sec.* J. TAFT, of Cincinnati.

*List of Societies represented in the American Dental Association.*

ALBANY DENTAL ASSOCIATION—Meets monthly at Albany. *Pres.* ROBERT NELSON, *Rec Sec.* W. F. WINNE, *Cor. Sec.* B. WOOD, of Albany.

BUFFALO DENTAL SOCIETY—Meets monthly at Buffalo. *Pres.* W. G. OLIVER, *Rec. Sec.* M. F. COOK.

BROOKLYN DENTAL ASSOCIATION—Meets semi-monthly *Pres.* W. C. PARKS, *Rec. Sec.* WM. C. HORNE, of Brooklyn.

CINCINNATI DENTAL SOCIETY—Meets semi-monthly at Cincinnati. *Pres.* Geo. WATT, *Rec. Sec.* Wm. TAFT.

CENTRAL OHIO DENTAL ASSOCIATION—Meets Semi-annually on the second Tuesday of May and November. *Pres.* Dr. M. DECAMP, Mansfield, O., *Sec.* M. A. SPENCER, Daylstown, O.

CENTRAL STATES DENTAL ASSOCIATION -Meets semi-annually. Next meeting at Indianapolis. The Annual meeting at Louisville, Ky., during the Holidays. The Semi-annual on the first Tuesday o April. *Pres.* Dr. J. A. McCLELLAND, *Sec.* J. F. CANINE, Louisville, Ky.

CENTRAL NEW YORK DENTAL ASSOCIATION. Meets semi-annually. *Pres.* S. B. PALMER, *Rec. Sec.* S. G. MARTIN, *Cor. Sec.* P. HARRIS of Skaneatles

CHICAGO DENTAL SOCIETY—Meets monthly at Chicago. *Pres.* S. B. NOBLE, *Rec. and Cor. Sec.* A. E. BROWN, of Chicago.

CONNECTICUT VALLEY DENTAL ASSOCIATION—Meets semi-annually, second Tuesday of May and October, *Pres.* J. BEALS. *Rec. Sec* L. D. SHEPHERD, of Boston. Mass.

CONNECTICUT STATE DENTAL ASSOCIATION—*Pres.* T. J. METCALF.

DELAWARE DENTAL ASSOCIATION—Meets semi-annually. *Pres* ——————. *Cor. Sec.* SAMUEL MARSHALL,

HUDSON RIVER ASSOCIATION OF DENTAL SURGEONS—Meets on the third Thursdays of May, September and January. *Pres.* E. D. FULLER, Peekskill. *Rec. Sec.* L. S. STRAW, Newburg.

HUDSON VALLEY DENTAL ASSOCIATION—meets monthly at Troy, N. Y. *Pres.* S. P. WELCH, Lansingburgh, *Rec. Sec.* E. J. YOUNG, *Cor. Sec.* L. C. WHEELER, of Troy.

ILLINOIS STATE DENTAL SOCIETY—Meets annually. *Pres.* H. N. LEWIS, QUINCY. *Sec* G. T. SMITH, Princeton, Ills.

INDIANA STATE DENTAL SOCIETY—Meets annually at Indianapolis on the last Tuesday of June. *Pres.* W. F. MORRILL, *Rec. and Cor. Sec* J. F. JOHNSON, of Indianapolis, Ind.

IOWA STATE DENTAL SOCIETY—Meets semi-annually Jan. and July. *Pres.* L. C. INGERSOLL. *Rec. Sec.* H. S. CHASE.

LAKE ERIE DENTAL SOCIETY.—Meets on the 24th Sept at Girard, Pa. *Pres.* A. B. ROBBINS

LEBANON VALLEY DENTAL ASSOCIATION—*Prest.* W. K. BRENIZER, *Sec.* S. H. GUILFORD.

LOUISVILLE DENTAL SOCIETY—Meets on the first Tuesday of each month. *Pres* ————, *Cor. Sec.* W. H. SHADOAN.

MAD RIVER VALLEY DENTAL SOCIETY—Meets quarterly. Next meeting in Xenia, on the third Monday of December next. *Prest.*, N. W. WILLIAMS, *Sec.*, G. L. PAINE.

MARYLAND ASSOCIATION OF DENTISTS—Meets the last Thursday of every month *Pres.* ROBT. ARTHUR.

MICHIGAN DENTAL SOCIETY—Meets annually second Tuesday of Oct. at Detroit. *Pres.* J. A, Harris, of Pontiac *Rec. and Cor. Sec.* GEO. LEARY, Adrian.

MISSISSIPPI VALLEY DENTAL ASSOCIATION—Meets annually in March at Cincinnati, *Pres.* W. H. GODDARD, *Rec. Sec.* G. W. FIELD, *Cor Sec.* C. M. WRIGHT, of Cin·

MISSOURI DENTAL ASSOCIATION—Meets on the first Tuesday of June, in St. Louis. *Pres.* Dr. H. J. McKellops. St. Louis, *Sec* H. Judd, St. Louis.

MERRIMAC VALLEY DENTAL SOCIETY—Meets semi annually first Tuesday of Oct, and May. *Pres.* A. Lawrence, *Rec. Sec.* G. A. Gerry, of Lowell.

MEMPHIS DENTAL SOCIETY.—Meets on the first Tuesday of each month. *Pres* G. W. Acree, *Sec.* L. W. Fletcher.

NASHVILLE DENTAL SOCIETY—Meets on the second Tuesday of each month. *Pres* W. H. Morgan, *Cor. Sec.* J. C. Russell

NEW YORK SOCIETY OF DENTAL SURGEONS—Meets semi-monthly in New York *Pres.* C. A. Marvin, *Rec. Sec.* C. E. Latimer, of New York.

NEW HAVEN DENTAL SOCIETY—Meets on the first Wednesday of each month at New Haven. *Pres.* J. T. Metcalf, *Rec. and Cor. Sec.* C. L. Smith, of New Haven.

NEW LONDON COUNTY DENTAL SOCIETY—Meets monthly.* *Pres.* R. W Brown *Rec. Sec.* Wm. Allender.

NORTHERN OHIO DENTAL ASSOCIATION—Meets Semi-annually, (first Tuesday o[illegible] May and October ) in Cleveland. *Pres.* B. F. Robinson, *Rec. Sec.* L. Buffett, *Cor. Sec* ——————Cleveland.

OHIO DENTAL COLLEGE ASSOCIATION—Meets annually in February at Cincinnati. *Pres.* W. H. Morgan, *Rec. Sec.* J Taft, of Cincinnati.

OHIO STATE DENTAL ASSOCIATION.—Meets semi-annually, at Columbus. [Next meeting—first Tuesday of December.] *Pres.* J. Taft, *Rec. Sec.* Geo.W. Keely, of Oxford.

ODONTOGRAPHIC SOCIETY OF PENNSYLVANIA—Meets monthly in Philadelphia *Pres.* Wm. C Head, *Rec. Sec.* T. C. Stellwagon, *Cor. Sec.* J. H. McQuillen, of Phila.

OLD COLONY DENTAL ASSOCIATION — Meets quarterly.* *Pres.* D. S. Dickerman, *Rec. Sec.* George R. Whitney, *Cor. Sec.* L. W. Puffer, North Bridgewater

PENNSYLVANIA ASSOCIATION OF DENTAL SURGEONS—Meets monthly in Phila *Pres.* W. W. Fouche, *Rec. Sec* James Truman *Cor. Sec.* G. W. Ellis, of Philadelphia.

PITTSBURG DENTAL ASSOCIATION—Meets monthly, (second Tuesday, in Pittsburg *Pres.* M. E. Gillespie, *Rec. Sec.* J. D White.

PENINSULAR DENTAL SOCIETY—Meets quarterly.* *Pres.* Samuel Marshall, *Rec. Sec.* W. G. A. Bonwill, *Cor. Sec.* S. S. Nones.

POUGHKEEPSIE DENTAL SOCIETY.—Meets monthly, *Pres.* J. H. Mann, *Sec.* H. F. Clark.

WESTERN DENTAL SOCIETY—Meets annually.* *Pres.* T. P. Abell, *Rec. Sec.* C. W Spalding, *Cor. Sec.* E. A Bogue.

ST. LOUIS DENTAL SOCIETY—Meets monthly at St. Louis.

WESTERN NEW YORK DENTAL ASSOCIATION—Meets semi-annually.* *Pres.* L. J. Walter, *Sec* W. C. Barrett, Warsaw, N. Y.

ST. LOUIS ODONTOLOGICAL SOCIETY—*Pres.* J. Paine, *Rec. Sec.* G. H. Silvers, *Cor Sec.* G. G. Samuel.

TENNESSEE DENTAL ASSOCIATION.—Meets semi-annually on the 20th of Dec. *Pres.* W. H. Morgan, *Sec.* W. T. Arrington, Memphis.

AMERICAN DENTAL CONVENTION—Meets annually first Tuesday of March, (next meeting at New York,) *Pres.* J G. Ambler, *Rec. Sec.* W. C. Horne. *Cor. Sec* J. S. Latimer, of New York.

**Place of meeting fixed by appointment.*

### CONTRIBUTORS.

W. H. ATKINSON, M. D., D. D. S.

B. WOOD, M. D.

W. H. SHADOAN, D. D. S.

WM. A. PEASE, D. D. S.

G. W. DE CAMP,

H. A. SMITH D. D. S.

GEO. B. SNOW, D. D. S.

WM. O. KULP.

A. S. TALBERT, D. D. S.

JOS. RICHARDSON, D. D. S.

A. LAWRENCE, D. D. S.

H. S. CHASE, D. D S.

R. J. POORE.

GEO. L. PAINE, D. D. S.

A. A. BLOUNT, M. D.

G A. MILLS.

C. W. SPALDING, D.D.S

J. DOUGLASS.

J. S LATIMER, D. D. S.

H. BENEDICT, D. D S.

S. D STEWART.

W. H GATES.

J. P. H. BROWN.

GEO. WATT, D. D. S.

J. TAFT, D. D S.

S. B. PALMER.

F. ABBOTT.

A. BERRY, D. D S.

A. W. MAXWELL, D. D. S.

H. F BISHOP.

JOSEPH S. HILDRETH M. D.

SAM'L JACKSON.

H. T. CRESSINGER.

J. CHESEBROUGH, D. D S.

W. G. REDMAN, D D. S.

L. D. SHEPARD, D. D. S.

### EXCHANGES,

ST. LOUIS MEDICAL AND SURGICAL JOURNAL,
BOSTON MEDICAL AND SURGICAL JOURNAL,
THE PACIFIC MEDICAL AND SURGICAL JOURNAL,
BUFFALO MEDICAL AND SURGICAL JOURNAL,
OHIO MEDICAL AND SURGICAL JOURNAL,
PHILADELPHIA MEDICAL AND SURGICAL REPORTER
CINCINNATI LANCET AND OBSERVER,
CHICAGO MEDICAL JOURNAL,
DENTAL COSMOS,
L'ART DENTAIRE,
THE LONDON DENTAL REVIEW,
BRAITHWAITE'S RETROSPECT,
SAN FRANCISCO MEDICAL PRESS,
THE DENTAL TIMES.
LADIES' REPOSITORY.
HALL'S JOURNAL OF HEALTH,
CHICAGO MEDICAL EXAMINER.
THE CINCINNATI JOURNAL OF MEDICINE.
MEDICAL RECORDER.
XENIA TORCHLIGHT.
THE U. S. MEDICAL AND SURGICAL JOURNAL.
THE DENTAL QUARTERLY.
THE DETROIT REVIEW.
SOUTHERN JOURNAL OF THE MEDICAL SCIENCES.
MEDICAL RECORD.
UNIVERSITY JOURNAL OF MEDICINE AND SURGERY,
NASHVILLE JOURNAL OF MEDICINE AND SURGERY,
MEDICAL REPORTER,
SOUTHERN MEDICAL AND SURGICAL JOURNAL,
AMERICAN ECLECTIC MEDICAL REVIEW.
AMERICAN JOURNAL OF DENTAL SCIENCE.
FARM AND FIRESIDE JOURNAL.

### CONTENTS.

### The Dental Register

OF THE WEST.

*Issued Monthly, at $3 a Year in Advance.*

DEVOTED TO THE INTERESTS OF THE DENTAL PROFESSION.

EDITED BY J. TAFT AND G. WATT.

The volume commences in January and closes in December.

The Register being issued monthly is always fresh, therefore indispensable to the practitioner who desires to keep posted up with the new inventions and improvements which are daily developed. Neither expense nor effort will be spared to give value and variety to its contents. Articles will be illustrated with well executed engravings whenever they will add to the interest or assist in explanation.

Its extensive circulation and frequency of issue makes it one of the BEST DENTAL ADVERTISING MEDIUMS in the United States.

RATES OF ADVERTISING.

One page, 1 year, $50. Half page, 1 year, $30. Quarter page, or card, 1 year $20.

All advertising bills payable in advance, or at furthest after the issue of the first number containing the advertisement. All transient advertisements to be paid for in advance.

☞ CONTRIBUTIONS SOLICITED.

Address, J. TAFT, or "DENTAL REGISTER," Cincinnati Ohio.

Business Communications to

J. TAFT, Publisher,

No. 238 Fourth St., between Plum and Central Avenue

